# Drug supply changes associated with a key policy on drug shortages in China: evidence from an interrupted time-series study

**DOI:** 10.3389/fphar.2026.1888875

**Published:** 2026-09-16

**Authors:** Shuchen Hu, Xingyu Liu, Xingchen Liu, Haoqi Wei, Xiaoyong Liu, Xingnan Tao, Fei Xue, Yu Fang, Caijun Yang

**Affiliations:** 1 Department of Pharmacy Administration, School of Pharmacy, Xi’an Jiaotong University, Xi’an, China; 2 School of Pharmaceutical Sciences, Zhengzhou University, Zhengzhou, China; 3 Center for Drug Safety and Policy Research, Xi’an Jiaotong University, Xi’an, China; 4 Xi’an No.3 Hospital, the Affiliated Hospital of Northwest University, Xi’an, China

**Keywords:** drug shortages, drug supply, interrupted time-series analysis, policy evaluation, China

## Abstract

**Background:**

Drug shortages pose significant challenges to healthcare systems globally, including China. In October 2019, China’s State Council launched a national strategy (D2019-47) to mitigate drug shortages. We aimed to characterize the supply status of medicines included in the National Drug Shortages List (NDSL), and assess changes associated with the local and national implementation periods of D2019-47.

**Methods:**

We conducted a retrospective interrupted time-series study using monthly procurement and delivery data from 31 centralized drug procurement service centers in China from April 2019 to June 2022. Fifty-two eligible molecule-formulation drug markets were included. Four periods were defined: pre-pandemic, COVID-19 lockdown, local implementation of D2019-47 (intervention 1), and national implementation of D2019-47 (intervention 2). Level and slope changes represented the immediate outcome change and the difference between post- and pre-intervention monthly trends, respectively. Outcomes were overall delivery rate (national supply status), worst-three delivery rate (supply status in the three lowest-supplied provinces), shortage frequency (shortage occurrences), and shortage coverage (geographic spread). Subgroup analyses were performed for currently resolved, price-driven (insufficient supply incentives), and production-driven shortages (manufacturing issues).

**Results:**

The mean monthly overall delivery rate was 87.3% in period 1, decreased to 78.5% in period 2, and increased to 89.6% in period 4, with similar patterns in other outcomes. Intervention 1 was associated with a level increase in overall delivery rate (4.7%, 95% CI: 2.4–7.1) and a subsequent slope increase (1.2%, 95% CI: 0.7–1.8), while the other indicators showed no significant changes. After Intervention 2, changes in slopes were mainly observed, including a decrease in overall delivery rate (−0.2%, 95% CI: −0.4 to 0.0), an increase in overall shortage frequency (4.1, 95% CI: 1.7–6.5), and an increase in overall shortage coverage (0.1, 95% CI: 0.1 to 0.1), with these changes observed primarily in price-driven markets.

**Conclusion:**

Overall delivery rates for NDSL medicines in China exceeded 75% from April 2019 to June 2022. Following intervention 1, the overall delivery rate improved, whereas most other aggregate shortage indicators showed no significant level or slope changes. Following intervention 2, additional improvement was limited, with adverse slope changes observed for several indicators. Regional disparities also persisted. These findings suggest that complementary strategies may be needed to sustain improvements and address persistent regional and market-specific supply vulnerabilities.

## 1 Background

Drug shortages posed an increasing challenge globally (World Health Organization, 2016), especially during the COVID-19 pandemic (Blankart and Felder, 2022). In recent years, shortages of various medications have been reported worldwide, including critical care medicines in Canada (Basky, 2020), antidepressants in South Africa (Mas et al., 2022), respiratory and cardiovascular medications in Europe (Hyde, 2020), and cefazolin in Japan (Honda et al., 2020). In the first quarter of 2024, active drug shortages in the United States reached a record high of 323 (ASHP, 2026). These shortages impose profound health impacts on healthcare systems, including dose delays, less effective treatments, increased morbidity, and even death (Phuong et al., 2019).

China confronted serious drug shortages during the last decade. Clinically essential agents, such as norepinephrine (Song et al., 2023), ibuprofen (Editorial. Where are the drugs?, 2023), oseltamivir (Editorial. Where are the drugs?, 2023), and phenobarbital (Li et al., 2024) have been reported to be in short supply. In response, China has actively implemented a series of strategies since 2017 (National Health Commission of China, 2017; The State Council of China, 2018; The State Council of China, 2019; National Health Commission of China, 2020a; National Health Commission of China, 2020b). Despite continuous policy efforts, evaluations of these measures remain inadequate—existing studies are limited in scope and timeliness. Therefore, it is urgent to assess changes associated with key policies using real-world data.

In October 2019, the State Council issued a national strategy (referred to as D2019-47) to ensure the supply and stable pricing of shortage drugs (The State Council of China, 2019)—the highest-level policy ever issued on this issue. D2019-47 proposed multiple measures, including multi-agency collaborative monitoring of drug shortages, stricter penalties for pharmaceutical monopolies, incentives for integrated production of active pharmaceutical ingredients (APIs) and finished drugs, and faster API review and approval. A key feature of D2019-47 is the list-based management approach: medicines in the National Drug Shortages List (NDSL) and Provincial Drug Shortages Lists (PDSL) are prioritized for additional measures, including expedited registration and approval, mandatory discontinuation reports by manufacturers, and a flexible procurement mechanism allowing more frequent price negotiations without a price ceiling.

The implementation of D2019-47 occurred in two stages. Initially, in October 2019, it was applied locally through PDSLs, as the NDSL had not yet been issued. With the release of the NDSL in December 2020 (National Health Commission of China, 2020b), the policy gained a defined nationwide scope, marking the transition to its national implementation. Notably, the nationwide lockdown following the COVID-19 outbreak in early 2020 (The State Council of China, 2020; BBC News, 2020) represented an acute external shock during the early implementation of D2019-47. To separate this disruption from the subsequent period, April 2020 was used as the operational cutoff for local implementation. Therefore, we sought to: (1) characterize the overall supply status of medicines in the National Drug Shortages List (NDSL) and characterize changes in their access during the COVID-19 pandemic; (2) assess changes in drug-shortage indicators associated with local (April 2020–March 2021) and national (April 2021–June 2022) implementations of D2019-47; (3) explore implications for shortage prevention policy.

## 2 Methods

We conducted a retrospective interrupted time-series study of procurement and delivery volumes for medicines listed in the National Drug Shortages List (NDSL).

### 2.1 Data sources and study cohort

We extracted pharmaceutical sales data from 31 provincial Centralized Drug Procurement Service Centers for the period between April 2019 to June 2022, using the name of drug molecule as the identifier. Monthly data were obtained for each product, including province, product name, manufacturer, molecule, dosage form (formulation), strength, volume ordered, volume delivered, and sales value. Volume ordered represented the amount requested by healthcare facilities, and volume delivered reflected the actual amount supplied.

We defined each unique drug molecule-formulation pair as an individual drug market, resulting in 58 drug markets. To ensure sufficient longitudinal coverage and geographic representation, we included markets with procurement data available for at least 26 months during the study period and with observations from at least 10 provinces per month. This process resulted in a final sample of 52 markets (molecule–formulation pairs) (Supplementary Appendix Table 1).

### 2.2 Indicators

For a market, the provincial monthly delivery rate (PMDR) was calculated as the ratio of delivered volume to ordered volume, capturing the gap between demand and supply. A drug market was considered to be experiencing a shortage in a given province if its PMDR was below 40%, as recommended by the National Healthcare Security Administration in an unpublished technical guideline for provincial monitoring of abnormal changes in drug supply.

Four monthly indicators were considered: (1) Overall Delivery Rate, representing the national supply status (Li and Sun, 2021; Lin et al., 2019; Wong et al., 2021; Tang et al., 2017), was the median of market medians, with each market median calculated across its provinces. (2) Worst-three delivery rate, representing the supply status in the worst-supplied three provinces (Zhao et al., 2020; Ni et al., 2020), was defined as the mean of market means, with each market mean calculated across its three lowest-supplied provinces. (3) Shortage Frequency, defined as shortage occurrences, was the total count of market–province combinations experiencing a shortage. (4) Shortage Coverage, was calculated as the mean number of provinces affected per market in a given month, indicating the geographic spread of drug shortages.

### 2.3 Setting

We defined four periods to assess policy-associated changes (Figure 1). Because no uniform implementation date was available, April 2020 and April 2021 were used as operational cutoffs for the ITS analysis rather than confirmed dates of policy implementation or effect onset.

1. Period 1: Pre-pandemic baseline (April 2019 – December 2019) – Served as the reference period before policy implementations.
2. Period 2: First wave of COVID-19 and nationwide lockdown (January–March 2020) – Captured changes in drug supply during the first wave of the pandemic.
3. Period 3: Local implementation of D2019-47 (April 2020 – March 2021) – April 2020 was used as the operational cutoff for Intervention 1. During the period, although the NDSL had not yet been issued, most PDSLs already covered the medicines later included in the NDSL.
4. Period 4: National implementation of D2019-47 (April 2021 – June 2022) – Following the release of the NDSL in December 2020 April 2021 was used as the operational cutoff for Intervention 2.

**FIGURE 1:**
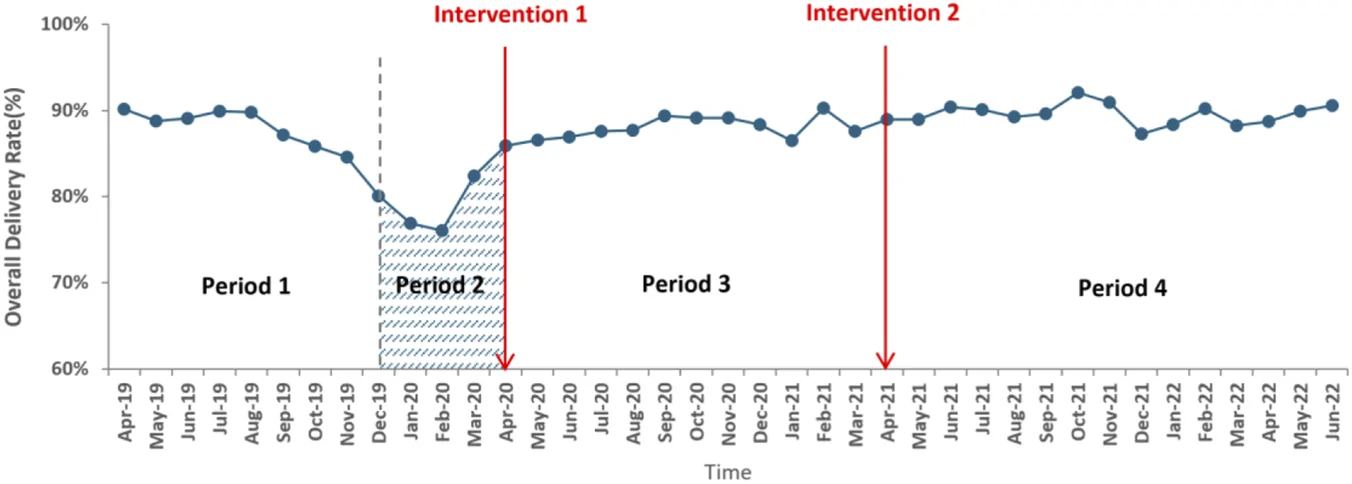
Study period definition and policy implementation stages.

### 2.4 Subgroup analysis

To further assess whether policy-associated changes differed across shortage categories, we conducted subgroup analyses. According to their shortage status or primary reported cause, 52 drug markets were classified into—currently resolved (8 markets), price-driven (15 markets), and production-driven (17 markets)—and one group with unknown causes (12 markets) (Supplementary Appendix Table 1):

1. Subgroup 1 (Currently resolved, 8 markets): Their shortage status was classified as resolved at the time of categorization.
2. Subgroup 2 (Price-driven, 15 markets): Shortages primarily caused by low prices and insufficient supply incentives.
3. Subgroup 3 (Production-driven, 17 markets): Shortages mainly attributable to manufacturing issues, such as API unavailability, quality risks, or factory relocation.

Subgroups 1–3 were analyzed, while markets with unknown causes were excluded from the subgroup analyses because their shortage drivers could not be classified.

### 2.5 Analysis strategy

Descriptive analyses were used to characterize drug-supply patterns across the four study periods; segmented regression was used to assess changes in the level and trend of the study indicators at the two operational cutoffs; and subgroup analyses were used to examine whether these changes differed across shortage categories. Two sensitivity analyses were conducted to assess the influence of the handling of Period 2 and uncertainty in intervention timing.

### 2.6 Interrupted time-series model

Interpretation of the segmented-regression estimates assumes that the preceding outcome trend would have continued in the absence of the interruption and that no other concurrent event caused changes at the same operational cutoffs. The interrupted time-series analysis covered period 1 (reference period), period 3 (intervention 1, local-implementation of D2019-47) and period 4 (intervention 2, national-implementation of D2019-47), excluding period 2. Period 2 was excluded because the initial COVID-19 outbreak constituted an acute external shock to drug supply. Including these observations in either the period one or the period 3 could obscure the estimation of policy-associated changes. Accordingly, changes following Intervention one were estimated relative to the pre-pandemic trend extrapolated to the operational cutoff, rather than as a continuous month-to-month transition from Period one to Period 3.

For each of the four monthly indicators, the segmented regression estimated: (1) baseline trends before the pandemic (period one trend); (2) the relative difference immediately following the two interruptions (change in level); (3) the difference in the trend between pre-and post-intervention (change in trend); and (4) the absolute trend during periods 3 and 4. The model specification is provided in the Appendix.

We undertook interrupted time-series analyses using the user-written ITSA command (Linden, 2015) within Stata version 17.0 (Stata Corp., College Station, United States of America). Residual autocorrelation was assessed using the Cumby–Huizinga test and autocorrelation plots, with Newey–West standard errors used for inference. Model diagnostics included *R* ^2^ and RMSE for goodness-of-fit and assessment of potential annual periodicity. Statistical uncertainty was quantified using 95% confidence intervals based on the Newey–West standard errors. This study follows the Strengthening the Reporting of Observational Studies in Epidemiology (STROBE) reporting guideline.

## 3 Results

### 3.1 Descriptive statistics

Table 1 shows that the mean monthly overall delivery rate was 87.3% in period 1 (pre-pandemic), decreased to 78.5% during the COVID-19 period (period 2), and increased to 89.6% in period 4 (after national implementation of D2019-47). Similarly, the worst-three delivery rate declined from 17.6% in period 1%–14.7% in period 2, and then increased to 34.4% in period 4. In line with this, the shortage frequency increased from 209.6 occurrences in period 1 to 243.0 in period 2, and then decreased substantially to 107.2 in period 4. The shortage coverage followed a similar pattern, increasing from 4.1 provinces in period one to 4.7 in period 2, and subsequently declining to 2.7 provinces in period 4.

**TABLE 1:** Descriptive statistics of study indicators by period, overall and by subgroup.

Groups	Periods	Indicators			
		Overall delivery rate mean (SD), %	Worst-three delivery rate mean (SD), %	Shortage frequency mean (SD), counts	Shortage coverage mean (SD), provinces
All	Period 1	87.3 (3.3)	17.6 (3.7)	209.6 (20.163)	4.1 (0.369)
	Period 2	78.5 (3.4)	14.7 (2.2)	243.0 (26.889)	4.7 (0.517)
	Period 3	87.9 (1.3)	28.2 (2.9)	145.2 (20.176)	3.1 (0.421)
	Period 4	89.6 (1.2)	34.4 (2.0)	107.2 (11.384)	2.7 (0.187)
Subgroup 1	Period 1	90.7 (3.5)	21.8 (2.6)	24.0 (2.449)	3.3 (0.498)
	Period 2	84.2 (3.9)	16.7 (1.6)	27.0 (3.606)	3.4 (0.451)
	Period 3	91.6 (2.2)	32.7 (3.6)	17.1 (2.314)	2.3 (0.326)
	Period 4	90.1 (1.9)	35.5 (3.7)	15.9 (3.907)	2.4 (0.521)
Subgroup 2	Period 1	87.1 (4.1)	18.2 (4.2)	59.7 (6.614)	4.0 (0.388)
	Period 2	78.7 (6.7)	16.1 (3.2)	72.0 (13.115)	4.8 (0.874)
	Period 3	87.2 (2.7)	29.2 (5.7)	39.2 (8.430)	2.8 (0.526)
	Period 4	89.3 (1.4)	34.1 (3.4)	30.3 (4.448)	2.6 (0.358)
Subgroup 3	Period 1	85.3 (2.8)	13.5 (5.3)	80.9 (10.647)	4.8 (0.635)
	Period 2	77.6 (3.9)	11.6 (2.3)	90.0 (3.606)	5.3 (0.212)
	Period 3	86.8 (1.9)	22.4 (3.3)	60.7 (9.099)	3.9 (0.537)
	Period 4	88.0 (3.3)	29.4 (3.5)	40.3 (8.672)	2.8 (0.517)

Period 1: April 2019 to December 2019; Period 2: January 2020 to March 2020; Period 3: April 2020 to March 2021; Period 4: April 2021 to June 2022. Values are presented as mean values for each period. The Shortage coverage is the average number of provinces affected per market per month and may be non-integer.

### 3.2 Overall delivery rate

Before the pandemic, the overall delivery rate was declining by 1.0% (95% CI: 1.5 to −0.5) per month, with a baseline of 91.4% (95% CI: 89.3–93.5) in April 2019 (Supplementary Appendix Table 2). Intervention 1 (the local implementation of D2019-47) was associated with an immediate level increase of 4.7% (95% CI: 2.4–7.1) in the overall delivery rate compared to the pre-pandemic period, and then increased by 0.2% (95% CI: 0.9–1.2) per month (Table 2; Figure 2a; Supplementary Appendix Table 2). After Intervention 2 (the national implementation), no significant level change was observed, while the slope decreased slightly by 0.2% (95% CI: −0.4 to 0.0), showing that the slope in Period 4 was close to zero (Table 2; Figure 2a; Supplementary Appendix Table 2). Across subgroups, Intervention one was associated with significant upward changes in both level and slope, whereas level and slope changes following Intervention 2 were largely non-significant, except for a significant downward change in slope in the price-driven subgroup (Subgroup 2: −0.7%, 95% CI: −1.0 to −0.4) (Supplementary Appendix Table 2; Supplementary Figure S1).

**TABLE 2:** Estimated coefficients of segmented regression model for overall delivery rate, worst-three delivery rate, shortage frequency and shortage coverage before and after two interventions, China, April 2019 to June 2022.

Indicators	Coefficient estimates (95% CI)			
​	Intervention 1	​	Intervention 2	​
​	Change in level	Change in slope	Change in level	Change in slope
Overall delivery rate (%)	4.7*** (2.4,7.1)	1.2*** (0.7,1.8)	0.4 (−0.7,1.6)	−0.2* (−0.4,0.0)
Worst-three delivery rate (%)	1.2 (−2.6,5.1)	−0.5 (−1.1,0.1)	1.9 (−1.4,5.2)	−0.7** (−1.1,-0.3)
Shortage frequency (counts)	−5.1 (−24.1,13.9)	1.8 (−0.9,4.5)	−2.1 (−20.8,16.6)	4.1** (1.7,6.5)
Shortage coverage (provinces)	0.1 (−0.3,0.4)	0.0 (0.0,0.1)	0.3* (0.0,0.6)	0.1*** (0.1,0.1)

* *P* < 0.05.

** *P* < 0.01.

*** *P* < 0.001.

**FIGURE 2:**
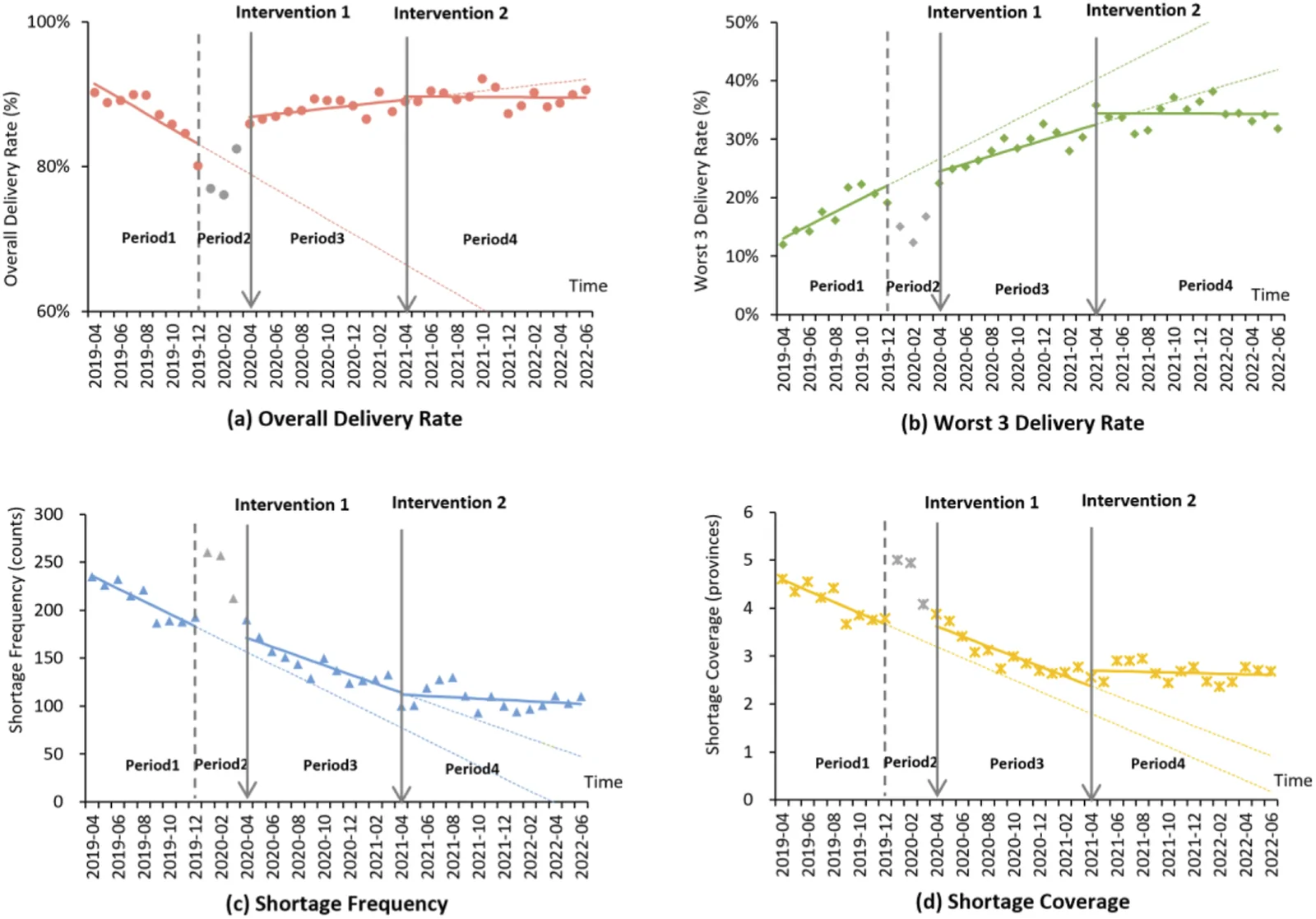
Trends and changes in indicators for all markets over study periods. Period 2 was not included in the interrupted time-series regression analysis. **(a–d)** show trends and changes for all study markets: **(a)** Overall delivery rate, **(b)** Worst-three delivery rate, **(c)** Shortage frequency, and **(d)** Shortage coverage.

### 3.3 Worst-three delivery rate

Before the pandemic, the worst-three delivery rate increased by 1.1% (95% CI: 0.6–1.6) per month, with a baseline of 13.1% (95% CI: 11.5–14.7) (Supplementary Appendix Table 2). Following Intervention 1, no significant overall level or slope change was observed, whereas in subgroups, a significant level increase was observed in Subgroup 1 (7.5%, 95% CI: 2.3–12.8) and a significant slope decrease in Subgroup 3 (−1.0%, 95% CI: −1.7 to −0.4) (Supplementary Appendix Table 2; Figure 2b). After Intervention 2, no significant level change was observed, whereas the slope decreased significantly by 0.7% (95% CI: −1.1 to −0.3), primarily driven by Subgroup 2 (−1.7%, 95% CI: −2.5 to −1.0) (Supplementary Appendix Table 2; Supplementary Figure S2).

### 3.4 Shortage frequency

Before the pandemic, shortage frequency declined by 6.6 cases per month (95% CI: −8.4 to −4.8), with a baseline of 236.0 (95% CI: 228.9–243.0) (Supplementary Appendix Table 2). Following Intervention 1, no significant overall level or slope change was observed, whereas in subgroups, Subgroup 1 showed a significant level decrease (−7.0, 95% CI: −12.4 to −1.6) and Subgroup 3 showed a significant level increase (9.5, 95% CI: 4.0–15.1) accompanied by a significant slope increase (1.5, 95% CI: 0.7–2.2) (Supplementary Appendix Table 2). After Intervention 2, no significant overall level change was observed, while the slope increased significantly (4.1, 95% CI: 1.7–6.5) Figure 2c, primarily driven by Subgroup 2 (2.2, 95% CI: 0.9–3.4) (Supplementary Appendix Table 2; Supplementary Figure S3).

### 3.5 Shortage coverage

Before the pandemic, the shortage coverage declined by 0.1 provinces per month (95% CI: −0.1 to −0.1), with a baseline of 4.6 (95% CI: 4.4–4.8) (Supplementary Appendix Table 2). Following Intervention 1, no significant overall changes were observed; however, Subgroup 3 showed a significant level increase (0.8, 95% CI: 0.2–1.2) accompanied by a slope rise (0.1, 95% CI: 0.0–0.2) (Supplementary Appendix Table 2; Supplementary Figure S4). After Intervention 2, overall level and slope increased (0.3, 95% CI: 0.0 to 0.6; 0.1, 95% CI: 0.1 to 0.1) Figure 2d, with significant slope increases in Subgroup 1 (0.1, 95% CI: 0.0–0.1) and Subgroup 2 (0.2, 95% CI: 0.1–0.2) (Supplementary Appendix Table 2; Supplementary Figure S4).

Additional model diagnostics did not materially alter the overall interpretation of the ITS findings (Supplementary Appendix Table 3), although some level-change estimates for the worst-three delivery rate were sensitive to periodic adjustment. Sensitivity analyses showed greater variation in the immediate level-change estimates across model specifications, including changes in direction for several estimates, whereas the directions of all slope-change estimates for the four overall outcomes were preserved (Supplementary Appendix Table 4).

## 4 Discussion

In this national interrupted time-series study, we assessed changes in the supply of NDSL medicines around the operational cutoffs for local and national implementation of D2019-47 and identified three key findings. First, monthly overall delivery rates for medications in the NDSL exceeded 75% in China from April 2019 to June 2022. Following the first operational cutoff, the overall delivery rate showed a significant upward level and slope change, whereas the other three overall indicators showed no significant level or slope changes. Following the second operational cutoff, most immediate level changes were not statistically significant, and several slope changes indicated limited further improvement or deterioration. Second, regional disparities persisted: although the worst-three delivery rate increased over the study period, it remained below 40%, indicating continued supply challenges in relatively low-supply provinces. Third, patterns of change differed across shortage categories, with price-driven and production-driven markets showing different temporal responses.

Overall, the supply of shortage medicines in China improved, with shortages significantly alleviated. Drug shortages have gradually shifted from systemic shortages to temporary and localized disruptions (National Health Commission of China, 2020b). These improvements coincided with the implementation of D2019-47 and a series of supporting measures (State Administration for Market Regulatio n, 2021; National Health Commission of China, 2022). Improvements were observed after the first operational cutoff, but subsequent trends leveled off over time. This pattern may reflect limitations in current policy strategies. Specifically, our previous analysis of policy texts from 2014 to 2024 revealed that current strategies primarily focus on shortage monitoring and response (Hu, 2025), rather than incentivizing sustained manufacturing. Especially for shortage drugs that require long-term production investment, current strategies may be insufficient to address these shortages. Additionally, other concurrent national events, market dynamics, and external disruptions—including the continuing effects of the COVID-19 pandemic (The BMJ Opinion, 2021; Das, 2021; Muhula et al., 2021) and geopolitical conflicts—may also have influenced the observed post-implementation trends.

Despite the overall improvement, regional disparities in drug supply persisted, with some areas experiencing more severe shortages. Improvements in national aggregate indicators may therefore mask persistent supply constraints in provinces with relatively low supply. The continued gap between the overall and worst-three delivery rates suggests that national-level improvements did not translate uniformly across provinces, reflecting regional inequities in drug access. These findings suggest that further efforts are needed to optimize cross-regional allocation and strengthen monitoring of low-supply regions.

D2019-47 included general measures applied to all drug markets (e.g., shortage monitoring and supply chain management), as well as targeted interventions addressing specific shortage categories. For markets classified as currently resolved, resolution at the time of categorization does not preclude future recurrence. Medicines with few manufacturers or limited therapeutic alternatives may remain vulnerable to renewed shortages and therefore warrant continued monitoring. Price-driven markets were addressed through procurement measures that removed price ceilings, whereas production-driven markets were addressed through prioritized approval processes intended to mitigate manufacturing constraints. This policy context may help explain the different response patterns observed across shortage categories.

Notably, the supply of price-driven drugs initially improved but subsequently deteriorated. One possible explanation for this pattern is rent-seeking behavior by suppliers under the price-incentive strategy. Rent-seeking refers to efforts to obtain privileged benefits created or protected by government policy rather than through productive activity (Aidt, 2016). In this context, some suppliers may deliberately restrict supply to maintain shortages despite having the capacity to meet demand, thereby retaining drugs on the NDSL and obtaining associated policy benefits, as listed drugs are eligible for procurement without a price ceiling. However, our study cannot directly establish the existence of such behavior, and this interpretation requires further investigation. Furthermore, we observed the disappearance of lower-priced specifications in the NDSL, while higher-priced alternatives emerged in the market. For instance, mitoxantrone injection was initially listed on the NDSL. Its 5 mL:5 mg specification was priced at approximately 40 RMB but disappeared from the market after the first quarter of 2020; in January 2022, a liposome injection (10 mL:10 mg) entered the market at a substantially higher price of 6988 RMB. Our previous global study also found that the drug price was not a significant predictor of shortages (Hu et al., 2025). This suggests that solely increasing prices may not effectively mitigate shortages, and this finding may inform policy evaluation in countries adopting similar policies, such as Saudi Arabia (Aljadeed et al., 2021) and Australia (Department of Healthand Aged Care, 2025).

Production-driven markets showed mixed responses to Intervention 1: the overall delivery rate improved, whereas the slope change in the worst-three delivery rate was negative and both shortage frequency and coverage increased. After Intervention 2, no significant level or slope changes were observed across the four indicators. Manufacturing difficulties and raw-material shortages are recognized as distinct causes of drug shortages, and mitigation strategies may need to be tailored to the underlying cause (Aronson et al., 2023). In a United States study of gastroenterology drug shortages, the median duration was 11.6 months for shortages attributed to manufacturing problems and 18.6 months for those attributed to raw-material constraints, compared with 8.6 months for supply–demand-related shortages (Patel et al., 2017). However, because our study did not directly measure manufacturing capacity, production investment, or API availability, the observed pattern should not be interpreted as evidence that manufacturing constraints caused the limited response.

### 4.1 Policy implications and recommendations

The persistent gap between the overall and worst-three delivery rates supports continued monitoring of provinces with relatively low supply and further evaluation of cross-regional allocation mechanisms. Given the plateauing post-implementation trends and different response patterns across shortage categories, three evidence-supported strategies may be considered.

1. Early notification and enforcement. Evidence from the United States and Canada indicates that mandated reporting of manufacturing interruptions can alleviate shortages, although its effectiveness varies with market competition (Lee et al., 2021). In eight European countries, only 5.2% of temporary shortage notifications were submitted at least 60 days in advance; in Finland, progressive notification fees were associated with earlier reporting (Ravela et al., 2023). These findings support combining early-notification requirements with mechanisms that promote compliance.
2. Storage obligations for selected essential medicines. A register study using data from Finland, Norway, and Sweden found that Finland’s storage-obligation system was associated with 45%–56% fewer shortage notifications and, in selected within-Finland comparisons, median shortage durations that were 14–21.5 days shorter (Ravela et al., 2025). Such obligations may provide a temporary buffer against production-related disruptions but should be adapted to local supply conditions.
3. Price incentives combined with failure-to-supply clauses. The later deterioration in price-driven markets suggests that price-based measures alone may be insufficient. A US supply-chain model estimated 25%–70% fewer shortages when a 30% price increase was combined with strengthened failure-to-supply clauses, whereas price increases alone had limited effects (Jia and Zhao, 2017). This supports evaluating price incentives together with contractual safeguards against failure to supply.

### 4.2 Strengths and limitations

Strengths of this study included the use of national-level data and of disaggregated analysis among several key subgroups. We analyzed drug supply trends across four periods covering 39 months, including the COVID-19 pandemic, which allowed us to assess whether gains in drug supply were sustained across the local and national implementation periods.

However, there were several limitations. First, the impact of the COVID-19 pandemic may have been underestimated, as we examined only medicines on the NDSL and excluded respiratory system drugs associated with the pandemic. Second, we may have underestimated changes associated with the local implementation of the D2019-47, as medications in the PDSLs were not examined. However, an existing study found that all 57 drugs in the NDSL were covered by the PDSLs in 24 provinces, indicating that the study can still capture policy-associated changes at the local level. Third, because no single uniform implementation date for D2019-47 was available, April 2020 and April 2021 were defined as operational cutoffs for the ITS analysis rather than confirmed dates of policy implementation or effect onset. This temporal uncertainty may have affected the estimates. In addition, although excluding Period 2 reduced contamination from the acute COVID-19 shock, pandemic-related recovery may have extended into the early months of Period 3; therefore, part of the estimated change following Intervention 1 may reflect post-pandemic recovery rather than changes associated with D2019-47. Finally, manufacturer-level data were not available; therefore, we could not directly verify the existence or underlying mechanisms of potential rent-seeking behavior. Further research using manufacturer-level data is needed to examine whether such behavior exists.

## 5 Conclusion

Overall delivery rates for medicines on the NDSL in China exceeded 75% from April 2019 to June 2022. The period following the first operational cutoff was associated with an improvement in overall delivery rate, whereas subsequent trends showed limited further improvement across several supply indicators. Regional disparities in drug supply also persisted. In price-driven markets, initial improvements were followed by later deterioration, suggesting that price-based measures alone may not ensure sustained supply. Further research is needed to clarify the mechanisms underlying these patterns and to identify complementary strategies for sustaining supply and reducing regional disparities.

## Data Availability

The raw data supporting the conclusions of this article will be made available by the authors, without undue reservation.
