# Supplementary material for: Drug supply changes associated with a key policy on drug shortages in China: evidence from an interrupted time-series study
Source: Front Pharmacol. 2026 Sep 16;17:1888875. doi: 10.3389/fphar.2026.1888875 (PMC13624402; doi:10.3389/fphar.2026.1888875)
Supplement: Supplementary file 1 [file DataSheet1.pdf]

Supplementary Material

1. eTable 1: Drug Markets, Shortage Causes, and Subgroup Classification

2. Model Specification for Interrupted Time Series Analysis (ITSA)

3. Interrupted Time Series Analyses

   eTable 2: Regression Coefficients of ITS Models for All Indicators and Subgroups

   eFigures 1 - 4: Trends of the Four Indicators by Subgroup

4. Model Diagnostics and Assessment of Annual Periodicity

   eTable 3: Goodness-of-Fit, Residual Autocorrelation, and Potential Annual Periodicity of the Interrupted Time-Series Models

5. Sensitivity Analyses

   eTable 4: Comparison of Estimated Level and Slope Changes in the Primary and Sensitivity Interrupted Time-Series Analyses

1. eTable 1: Drug Markets, Shortage Causes, and Subgroup Classification

eTable 1: Drug Markets, Shortage Causes, and Subgroup Classification

No.	Subgroup	Markets	Shortage Causes
1	Subgroup1	Ethacridine-Parenteral	Resolved
2		Dopamine-Parenteral	Resolved
3		Dobutamine-Parenteral	Resolved
4		Thiamazole-Oral	Resolved
5		Norepinephrine-Parenteral	Resolved
6		Penicillamine-Oral	Resolved
7		Mitomycin-Parenteral	Resolved
8		Tretinoin-Oral	Resolved
9	Subgroup2	Benzathine Benzylpenicillin-Parenteral	Price-driven
10		Bleomycin A5-Parenteral	Price-driven
11		Diazepam-Parenteral	Price-driven
12		Phentolamine-Parenteral	Price-driven
13		Furosemide-Parenteral	Price-driven
14		Mannitol-Parenteral	Price-driven
15		Magnesium Sulfate-Parenteral	Price-driven
16		Mercaptopurine-Oral	Price-driven
17		Naloxone-Parenteral	Price-driven
18		Thrombin-Topical	Price-driven
19		Neostigmine Methylsulfate-Parenteral	Price-driven
20		Propafenone-Parenteral	Price-driven
21		Hydrocortisone-Parenteral	Price-driven
22		Sodium Nitroprusside-Parenteral	Price-driven
23		Vitamin K1-Parenteral	Price-driven
24	Subgroup3	Allopurinol-Oral	Production-driven
25		Cytarabine-Parenteral	Production-driven
26		Amiodarone-Parenteral	Production-driven
27		Bleomycin-Parenteral	Production-driven
28		Cyclophosphamide-Parenteral	Production-driven
29		Digoxin-Oral	Production-driven
30		Sodium Dimercaptopropanesulfonate-Parenteral	Production-driven
31		Methotrexate-Parenteral	Production-driven
32		Lobeline-Parenteral	Production-driven
33		Urokinase-Parenteral	Production-driven
34		Posterior Pituitary-Parenteral	Production-driven
35		Oxytocin-Parenteral	Production-driven
36		Nitroglycerin-Oral	Production-driven
37		Nitroglycerin-Parenteral	Production-driven
38		Etoposide-Parenteral	Production-driven

No.	Subgroup	Market	Shortage Causes
39	Subgroup3	Protamine-Parenteral	Production-driven
40		Sodium Thiosulfate-Parenteral	Production-driven
41	Subgroup4	Atropine-Parenteral	Unknown
42		Arginine-Parenteral	Unknown
43		Adrenocorticotropin (ACTH)-Parenteral	Unknown
44		Deslanoside-Parenteral	Unknown
45		Pralidoxime Chloride-Parenteral	Unknown
46		Nikethamide-Parenteral	Unknown
47		Epinephrine-Parenteral	Unknown
48		Isoprenaline-Parenteral	Unknown
49		Methylthioninium Chloride-Parenteral	Unknown
50		Verapamil-Parenteral	Unknown
51		Vincristine-Parenteral	Unknown
52		Calcium Gluconate-Parenteral	Unknown

Note: Nos. 36 and 37 both refer to nitroglycerin, the same chemical with oral and parenteral routes, and are therefore defined as two separate markets.

2. Model Specification for Interrupted Time Series Analysis (ITSA)

Three periods were included in the ITS analysis: the pre-pandemic period before policy implementation (Period 1), the local implementation of D2019-47 (Period 3), and the national implementation of D2019-47 (Period 4), excluding the nationwide COVID-19 lockdown period (Period 2). The analysis estimated baseline trends, immediate level changes, and trend changes following the interventions for each of the four key indicators using separate ITS regressions. Taking the overall delivery rate as an example, the ITS regression model is specified as follows:

$$Y_{it} = \beta_0 + \beta_1 T_{it} + \beta_2 D_{1i} + \beta_3 D_{2i} + \beta_4 (T_{it} \times D_{1i}) + \beta_5 (T_{it} \times D_{2i}) + \epsilon_{it}$$

where Y_{it} represents the overall delivery rate at time t. β_0 is the baseline level (intercept), representing the expected value of the outcome at the start of the series, before any interventions. $\beta_1 T_{it}$ represents the pre-intervention trend, with T_{it} as a continuous time variable. D_{1i} and D_{2i} are dummy variables for Intervention 1 (local implementation) and Intervention 2 (national implementation), respectively. D_{1i} is coded as "0" before the Intervention 1 and "1" afterwards, while D_{2i} is coded as "0" before the Intervention 2, including the period after Intervention 1, and "1" afterwards. β_2 and β_3 indicate the immediate level change after Interventions 1 and 2. The interaction terms $T_{it} \times D_{1i}$ and $T_{it} \times D_{2i}$ capture changes in trend after each intervention, represented by β_4 and β_5. ϵ_{it} is the error term.

All analyses were performed using Stata 17.0 and Microsoft Excel 2019. $P < 0.05$ was considered statistically significant.

3. Interrupted Time Series Analyses

eTable 2: Regression Coefficients of ITS Models for All Indicators and Subgroups

	Coefficient estimates (95% CI)							
	Baseline level	Trends in three periods			Intervention 1		Intervention 2	
		Period 1	Period 3	Period 4	Change in level	Change in slope	Change in level	Change in slope
**Overall Delivery Rate (%)**								
**All**	91.4	-1.0	0.2	0.0	4.7***	1.2***	0.4	-0.2*
	(89.3，93.5)	(-1.5，-0.5)	(0.0，0.4)	(-0.1，0.1)	(2.4，7.1)	(0.7，1.8)	(-0.7，1.6)	(-0.4，0.0)
**Subgroup 1**	95.2	-1.1	0.1	-0.1	6.2***	1.2***	-1.5	-0.1
	(93.3，97.1)	(-1.6，-0.7)	(-0.2，0.3)	(-0.2，0.1)	(4.2，8.3)	(0.6，1.7)	(-3.2，0.3)	(-0.4，0.2)
**Subgroup 2**	92.7	-1.4	0.6	-0.1	3.9*	2.0***	-1.1	-0.7***
	(91.6，93.8)	(-1.8，-1.0)	(0.3，0.8)	(-0.3，0.1)	(0.7，7.1)	(1.5，2.5)	(-3.6，1.4)	(-1.0，-0.4)
**Subgroup 3**	88.0	-0.7	0.2	0.1	3.5*	0.9***	-1.1	-0.1
	(85.9，90.1)	(-1.1，-0.2)	(0.1，0.4)	(-0.2，0.4)	(0.6，6.5)	(0.5，1.4)	(-3.6，1.3)	(-0.4，0.2)
**Worst 3 Delivery Rate (%)**								
**All**	13.1	1.1	0.7	0.0	1.2	-0.5	1.9	-0.7**
	(11.5，14.7)	(0.6，1.6)	(0.3，1.0)	(-0.2，0.2)	(-2.6，5.1)	(-1.1，0.1)	(-1.4，5.2)	(-1.1，-0.3)
**Subgroup 1**	19.3	0.6	0.0	0.4	7.5**	-0.6	0.2	0.3
	(17.2，21.3)	(0.3，1.0)	(-0.7，0.8)	(0.0，0.7)	(2.3，12.8)	(-1.5，0.2)	(-5.9，6.3)	(-0.5，1.2)
**Subgroup 2**	14.5	0.9	1.2	-0.5	-0.3	0.3	0.4	-1.7***
	(11.9，17.2)	(0.1，1.7)	(0.6，1.9)	(-0.8，-0.2)	(-7.1，6.5)	(-0.7，1.4)	(-5.5，6.3)	(-2.5，-1.0)
**Subgroup 3**	6.8	1.7	0.7	0.6	-3.2	-1.0**	-1.4	-0.1
	(4.7，8.8)	(1.1，2.2)	(0.3，1.0)	(0.3，0.9)	(-7.8，1.4)	(-1.7，-0.4)	(-5.4，2.5)	(-0.5，0.3)
**Shortage Frequency (counts)**								
**All**	236.0	-6.6	-4.8	-0.7	-5.1	1.8	-2.1	4.1**
	(228.9，243.0)	(-8.4，-4.8)	(-6.8，-2.7)	(-2.0，0.6)	(-24.1，13.9)	(-0.9，4.5)	(-20.8，16.6)	(1.7，6.5)
**Subgroup 1**	24.0	0.0	0.0	-0.2	-7.0*	0.0	0.2	-0.2
	(21.6，26.4)	(-0.7，0.7)	(-0.4，0.5)	(-0.6，0.1)	(-12.4，-1.6)	(-0.8，0.8)	(-4.7，5.1)	(-0.8，0.3)
**Subgroup 2**	64.7	-1.3	-1.7	0.4	-4.6	-0.5	-0.6	2.2**
	(60.0，69.5)	(-2.8，0.3)	(-2.9，-0.5)	(0.0，0.9)	(-17.9，8.7)	(-2.5，1.5)	(-9.3，8.2)	(0.9，3.4)
**Subgroup 3**	95.3	-3.6	-2.1	-1.4	9.5**	1.5***	3.3	0.7
	(92.0，98.5)	(-4.2，-3.0)	(-2.6，-1.6)	(-2.1，-0.7)	(4.0，15.1)	(0.7，2.2)	(-5.8，12.4)	(-0.1，1.6)
**Shortage Coverage (provinces)**								
**All**	4.6	-0.1	-0.1	0.0	0.1	0.0	0.3*	0.1***
	(4.4，4.8)	(-0.1，-0.1)	(-0.1，-0.1)	(0.0，0.0)	(-0.3，0.4)	(0.0，0.1)	(0.0，0.6)	(0.1，0.1)
**Subgroup 1**	3.2	0.0	0.0	0.0	-0.8	-0.1	0.3	0.1*
	(2.8，3.6)	(-0.1，0.1)	(-0.1，0.0)	(0.0，0.1)	(-1.7，0.1)	(-0.2，0.0)	(-0.4，1.0)	(0.0，0.1)
**Subgroup 2**	4.3	-0.1	-0.1	0.0	-0.3	0.0	0.1	0.2***
	(4.0，4.6)	(-0.2，0.0)	(-0.2，0.0)	(0.0，0.1)	(-1.0，0.5)	(-0.2，0.1)	(-0.4，0.7)	(0.1，0.2)
**Subgroup 3**	5.6	-0.2	-0.1	-0.1	0.8**	0.1**	0.4	0.0
	(5.4，5.8)	(-0.2，-0.2)	(-0.2，-0.1)	(-0.1，-0.1)	(0.3，1.2)	(0.0，0.2)	(-0.1，0.8)	(0.0，0.1)

Note: The baseline level represents the estimated outcome of intercept. Trends in the three periods indicate the monthly slope during each period. The periods are defined as follows: Period 1 = pre-pandemic, Period 3 = local implementation (Intervention 1), and Period 4 = national implementation (Intervention 2). *P < 0.05, **P < 0.01, ***P < 0.001.

eFigure 1: Trends and changes in overall drug delivery rate over study periods

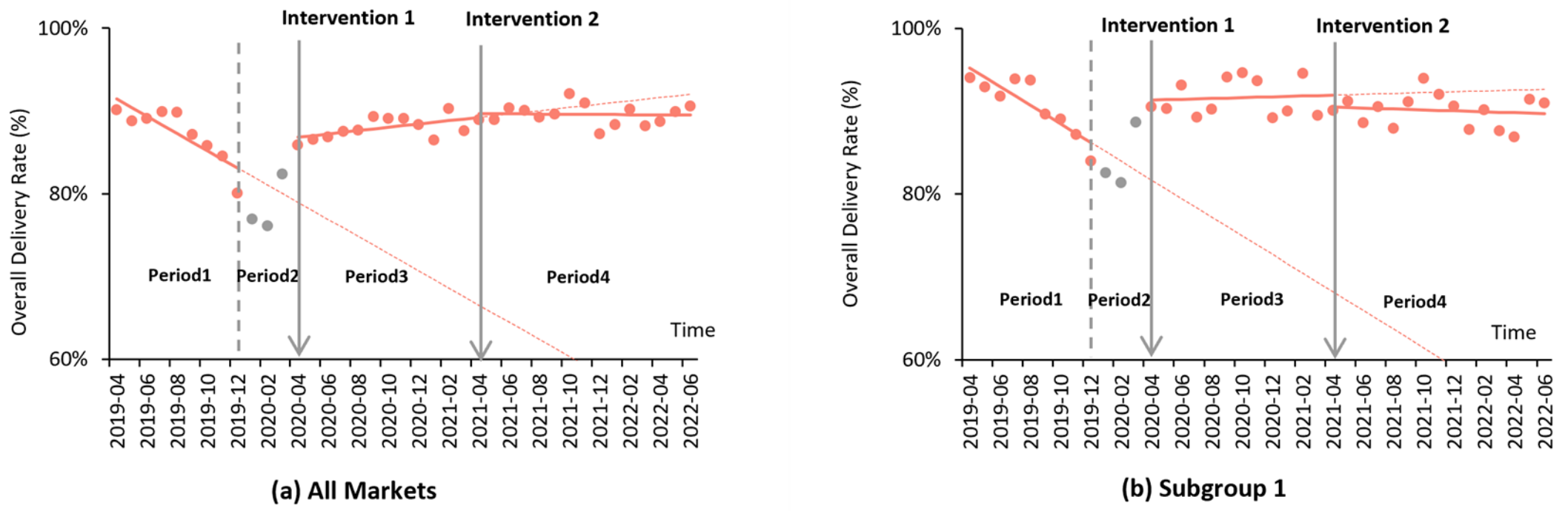

eFigure 2: Trends and changes in Worst 3 Delivery Rate over study periods

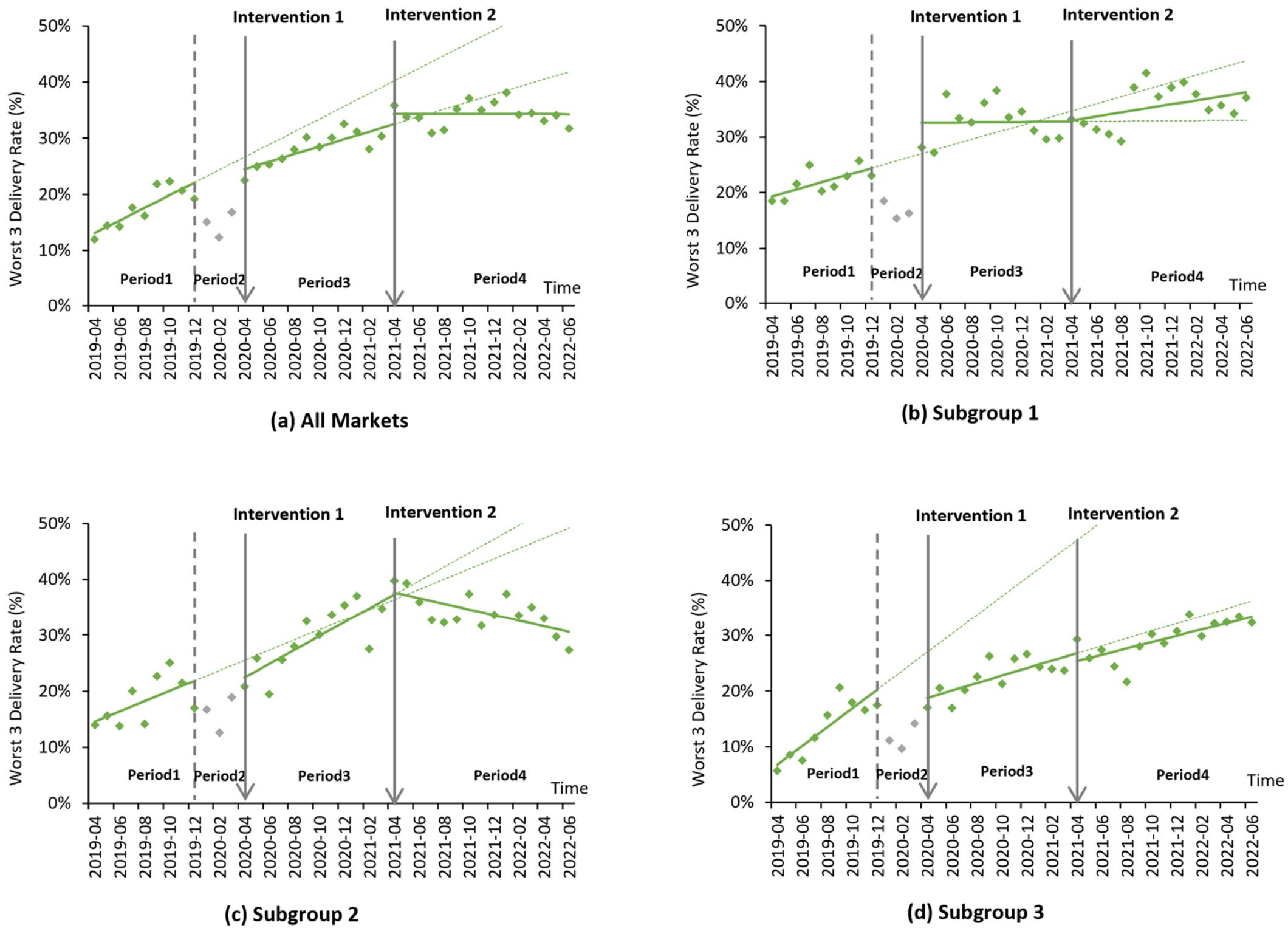

eFigure 3: Trends and changes in Shortage Frequency over study periods

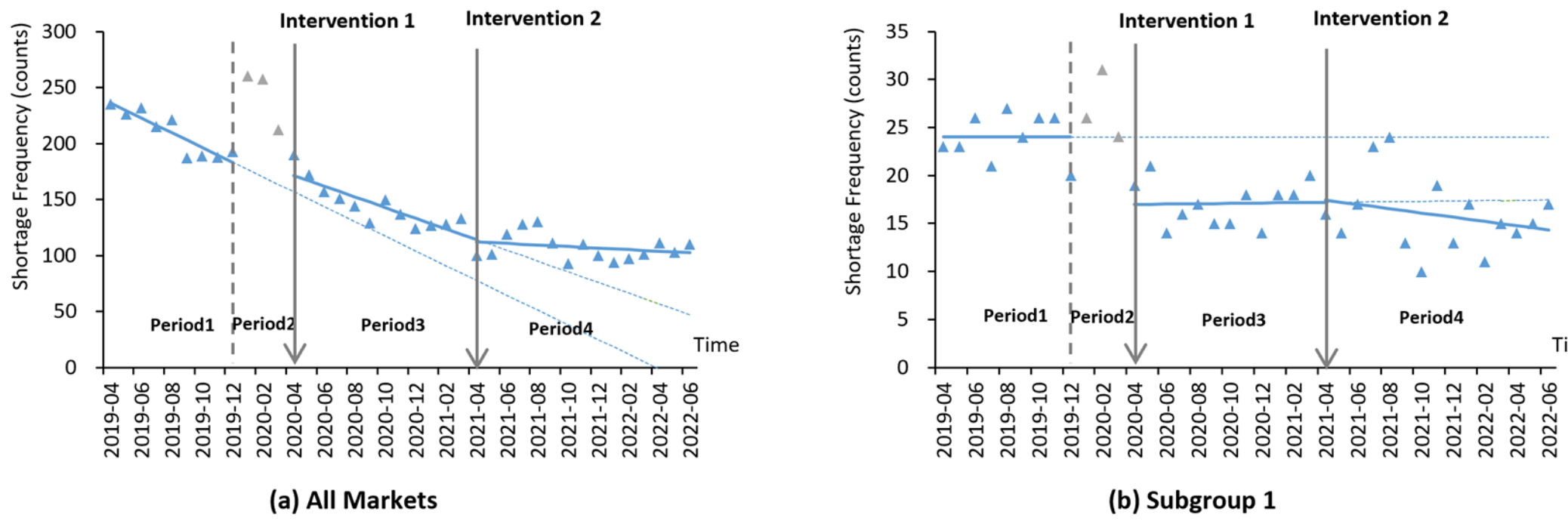

eFigure 4: Trends and changes in Shortage Coverage over study periods

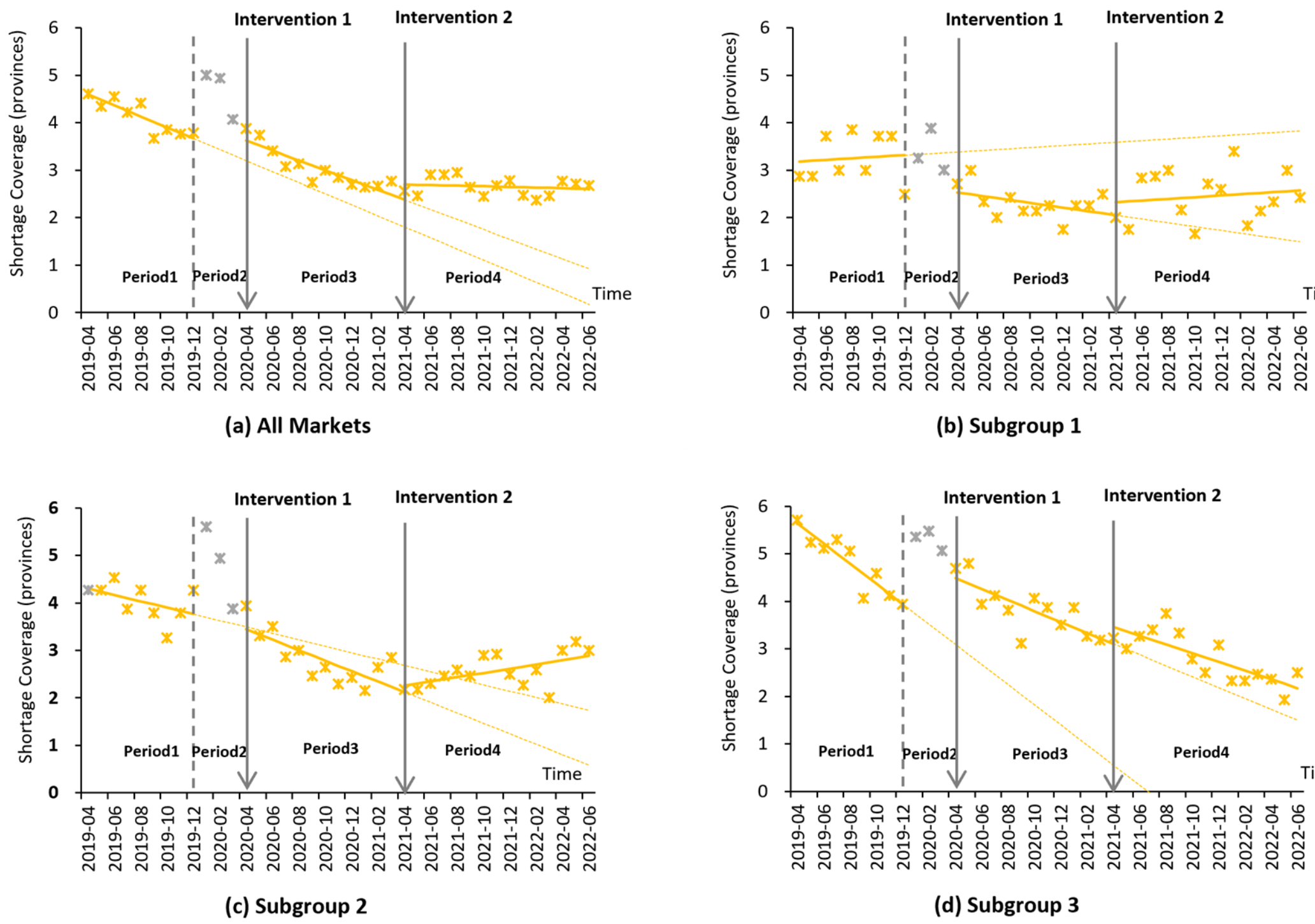

Note: Period 2 was not included in the interrupted time-series regression analysis. Panel (a) presents the results for all study markets; panel (b) presents the results for Subgroup 1 (Resolved); panel (c) presents the results for Subgroup 2 (Price-driven shortages); and panel (d) presents the results for Subgroup 3 (Production-driven shortages).

4. Model Diagnostics and Assessment of Annual Periodicity

eTable 3: Goodness-of-Fit, Residual Autocorrelation, and Potential Annual Periodicity of the Interrupted Time-Series Models

Outcome	Analysis	NW lag	R^2	RMSE	CH *P*, lags 1–12	Annual periodicity *P*	Sensitivity to periodic adjustment
Overall delivery rate	Overall	6	0.641	0.014	0.067	<0.001	Generally consistent
	Currently resolved	6	0.421	0.021	0.020	0.008	Generally consistent
	Price-driven	0	0.759	0.015	0.236	0.021	Generally consistent
	Production-driven	4	0.284	0.026	0.258	0.206	Not applicable
Worst-three delivery rate	Overall	0	0.935	0.020	0.092	<0.001	Some level estimates sensitive
	Currently resolved	1	0.781	0.033	0.066	<0.001	Generally consistent; magnitude changed
	Price-driven	0	0.846	0.033	0.582	0.105	Not applicable
	Production-driven	0	0.903	0.025	0.441	0.218	Not applicable
Shortage frequency	Overall	0	0.949	10.843	0.331	0.061	Not applicable
	Currently resolved	0	0.574	3.198	0.504	0.318	Not applicable
	Price-driven	0	0.869	5.251	0.615	0.461	Not applicable
	Production-driven	0	0.928	5.462	0.533	0.334	Not applicable
Shortage coverage	Overall	0	0.928	0.196	0.359	0.148	Not applicable
	Currently resolved	3	0.452	0.470	0.186	0.710	Not applicable
	Price-driven	0	0.815	0.340	0.541	0.328	Not applicable
	Production-driven	0	0.903	0.326	0.408	0.940	Not applicable

Note: NW, Newey–West; CH, Cumby–Huizinga. CH P values are from the joint test for residual autocorrelation across lags 1–12. Newey–West lag lengths were informed by autocorrelation diagnostics through lag 6 in the primary analysis. Potential annual periodicity was assessed by jointly testing 12-month sine and cosine terms. Models with evidence of potential annual periodicity ($P<0.05$) were additionally refitted with these terms to assess the sensitivity of intervention-associated level and slope estimates. Not applicable indicates that periodic adjustment was not performed because no evidence of potential annual periodicity was detected.

5. Sensitivity Analyses

Two sensitivity analyses were conducted to assess the influence of the treatment of the acute COVID-19 period and uncertainty in intervention timing on the ITS estimates. **Sensitivity analysis 1 (SA1)** retained the complete 39-month series, including January–March 2020, and included a temporary COVID-19 indicator (coded as 1 for January – March 2020 and 0 otherwise), while retaining the primary operational cutoffs of April 2020 and April 2021. **Sensitivity analysis 2 (SA2)** likewise retained the complete series and the COVID-19 indicator but used November 2019 and January 2021 as alternative calendar-aligned cutoffs, corresponding to the first month after the issuance of D2019-47 and the release of the NDSL, respectively. Outcome-specific Newey – West lag specifications were unchanged across analyses.

Across the primary analysis, SA1, and SA2, variation was concentrated mainly in the immediate level-change estimates and, in some cases, their statistical significance; several level-change estimates also changed direction. In contrast, the direction of all slope-change estimates was preserved across both sensitivity analyses. For Intervention 2, some sensitivity specifications produced negative immediate level changes in the delivery-rate outcomes, indicating that the observed outcome level at the assumed intervention point was below that projected from the preceding trend. Full estimates and 95% confidence intervals are presented in eTable 4.

eTable 4: Comparison of Estimated Level and Slope Changes in the Primary and Sensitivity Interrupted Time-Series Analyses

Outcome	Intervention	Change	Primary analysis	Sensitivity Analysis 1	Sensitivity Analysis 2
**Overall delivery rate (%)**	Intervention 1	Level change	4.7***<br>(2.4, 7.1)	6.9***<br>(3.3, 10.4)	−3.9***<br>(−5.5, −2.2)
		Slope change	1.2***<br>(0.7, 1.8)	1.1***<br>(0.7, 1.5)	1.1***<br>(0.7, 1.5)
	Intervention 2	Level change	0.4<br>(−0.7, 1.6)	0.4<br>(−0.8, 1.6)	−1.8**<br>(−3.0, −0.6)
		Slope change	−0.2*<br>(−0.4, 0.0)	−0.2*<br>(−0.4, 0.0)	−0.5***<br>(−0.7, −0.3)
**Worst-three delivery rate (%)**	Intervention 1	Level change	1.2<br>(−2.6, 5.1)	−2.1<br>(−7.2, 3.1)	−4.7***<br>(−7.4, −2.0)
		Slope change	−0.5<br>(−1.1, 0.1)	−0.5<br>(−1.0, 0.1)	−0.7***<br>(−1.1, −0.3)
	Intervention 2	Level change	1.9<br>(−1.4, 5.2)	1.9<br>(−1.4, 5.2)	−0.6<br>(−3.4, 2.1)
		Slope change	−0.7***<br>(−1.1, −0.3)	−0.7***<br>(−1.1, −0.3)	−0.8***<br>(−1.1, −0.5)
**Shortage frequency (counts)**	Intervention 1	Level change	−5.081<br>(−24.070, 13.907)	19.209<br>(−6.110, 44.528)	16.718<br>(−2.760, 36.197)
		Slope change	1.817<br>(−0.910, 4.543)	2.378<br>(−0.660, 5.416)	2.532<br>(−0.433, 5.496)
	Intervention 2	Level change	−2.076<br>(−20.774, 16.623)	−2.076<br>(−20.920, 16.768)	1.655<br>(−13.323, 16.634)
		Slope change	4.098***<br>(1.681, 6.514)	4.098**<br>(1.662, 6.533)	4.164***<br>(2.461, 5.867)
**Shortage coverage (provinces)**	Intervention 1	Level change	0.071<br>(−0.251, 0.393)	0.509*<br>(0.084, 0.934)	0.312<br>(−0.153, 0.776)
		Slope change	0.013<br>(−0.034, 0.060)	0.024<br>(−0.029, 0.077)	0.035<br>(−0.033, 0.102)
	Intervention 2	Level change	0.325*<br>(0.003, 0.647)	0.325*<br>(0.0004, 0.649)	0.073<br>(−0.145, 0.291)
		Slope change	0.097***<br>(0.054, 0.140)	0.097***<br>(0.054, 0.140)	0.093***<br>(0.061, 0.126)

Note：*$P < 0.05$, **$P < 0.01$, ***$P < 0.001$.
